# Predisposing conditions for bacterial meningitis in children: what radiologists need to know

**DOI:** 10.1007/s11604-021-01191-9

**Published:** 2021-08-25

**Authors:** Sota Masuoka, Osamu Miyazaki, Hiroaki Takahashi, Yoshiyuki Tsutsumi, Takashi Hiyama, Masayuki Kitamura, Reiko Okamoto, Mikiko Miyasaka, Manabu Minami, Shunsuke Nosaka

**Affiliations:** 1 Department of Diagnostic Radiology, National Center for Child Health and Development, 2-10-1 Okura, Setagaya-ku, Tokyo, 157-8535 Japan; 2 Department of Diagnostic Radiology, Mayo Clinic Minnesota, 200 First St. SW, Rochester, MN 55905 USA; 3 Department of Diagnostic Radiology, National Cancer Center Hospital East, 6-5-1 Kashiwanoha, Kashiwa, Chiba, 277-8577 Japan; 4 Department of Diagnostic and Interventional Radiology, Faculty of Medicine, University of Tsukuba, 1-1-1, Tennoudai, Tsukuba, Ibaraki 305-8575 Japan

**Keywords:** Pediatrics, Bacterial infections, Meningitis, Multidetector computed tomography, Magnetic resonance imaging

## Abstract

A variety of underlying diseases can predispose infants and children to bacterial meningitis (BM). For the diagnosis, treatment, and prevention of its recurrence, radiologists should be familiar with its predisposing conditions so that they can suggest the appropriate imaging approach. Predisposing conditions of BM can be broadly classified into two categories: infection spread from the adjacent tissue to the cerebrospinal fluid (CSF) space and immunodeficiency. Diseases in the former category are further divided according to regardless of whether there is a structural defect between the CSF space and the adjacent tissue. When a structural defect is suspected in a patient with BM, computed tomography (CT) of the head and magnetic resonance (MR) imaging are first-line imaging examinations. Radionuclide cisternography should be implemented as a second-line step to identify the CSF leak site. In patients with suspected parameningeal infection without any structural defect, such as sinusitis or otitis media/mastoiditis, CT or MR images can identify not only the disease itself but also the associated intracranial complications. The purpose of this article is to discuss the diagnostic approach and imaging findings associated with the variety of conditions predisposing patients to recurrent BM, focusing on the role of radiology in their management.

## Introduction

Bacterial meningitis (BM) is an inflammation of the meninges (affecting the pia, arachnoid, and subarachnoid space) in response to a bacterial infection [1]. It is a medical emergency associated with a high mortality rate and incidence of neurological sequelae, if untreated. Thus, early diagnosis and the prompt initiation of empirical antimicrobial treatment are crucial. Popular causative organisms in children include *Haemophilus influenzae type b* (Hib), *Streptococcus pneumoniae*, and *Neisseria meningitides*. The widespread use of the Hib conjugate vaccine and the heptavalent pneumococcal conjugate vaccine (PCV7) has virtually eradicated meningitis caused by these organisms in many high-income countries [1]. As a result, the prevalence of bacterial meningitis in patients with predisposing conditions such as anatomical abnormalities, has increased. Recurrent BM is defined as a second episode of meningitis caused by a different pathogen than the one in the prior infection or occurring more than 3 weeks after the resolution of the prior episode [2, 3]. Approximately 75% of the cases of BM occur in children under 5 years of age mainly due to the immature development of their immune system [1, 4–6]. A total of 1.3% of children with BM who underwent inpatient care had a recurrent episode [7, 8]. A predisposing condition for recurrent BM could be identified in 39% of patients [3]. Early diagnosis of these predisposing conditions is crucial for preventing recurrence of BM and improving the prognosis of the affected individuals. Therefore, the role of the radiological examination is critical in searching for these predisposing conditions in addition to assessing the complications of BM. In this article, the classification of the predisposing conditions for recurrent BM in children is described, along with the diagnostic approaches for each disorder.

## Management of children with suspected BM

**Fig. 1:**
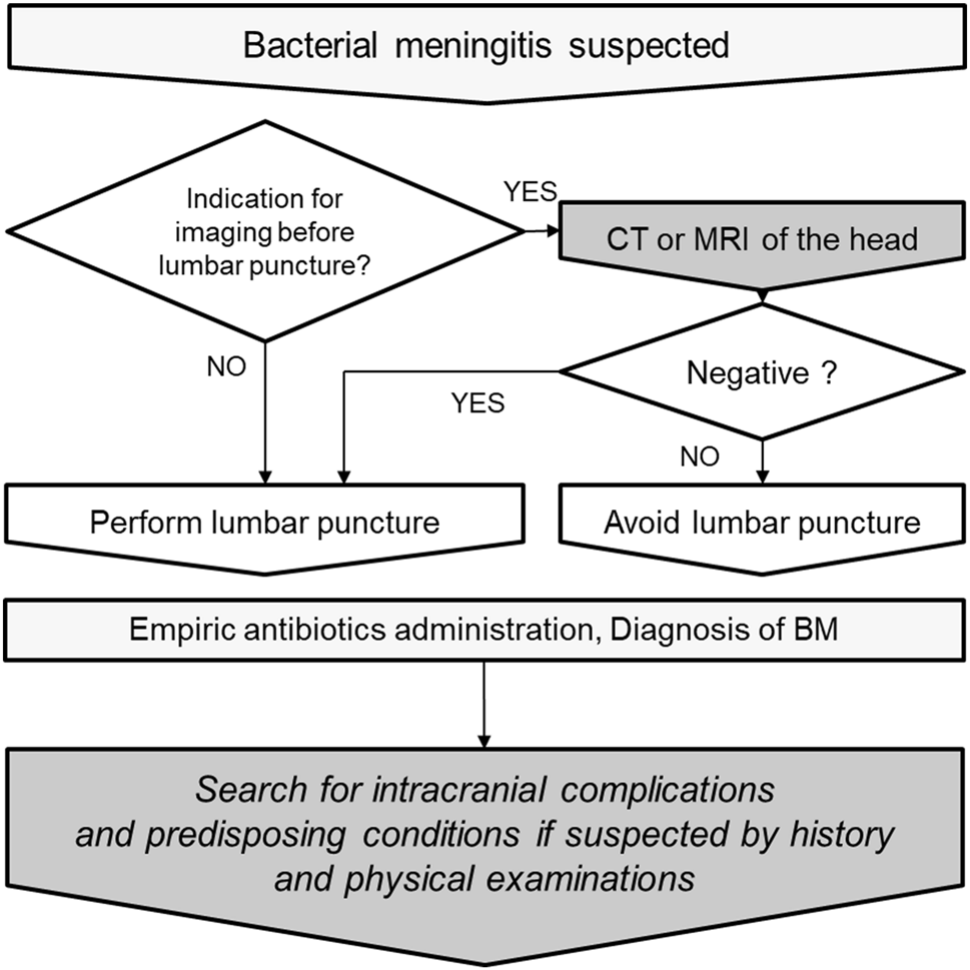
The management algorithm of the initial evaluation for suspected bacterial meningitis (BM). Cerebrospinal fluid analysis by lumbar puncture enables the confirmation of BM, and computed tomography (CT) or magnetic resonance (MR) images of the head are primarily used to confirm existing conditions that could be contraindications for a lumbar puncture (i.e., a central nervous system mass, Chiari malformation type I, or another cause of increased intracranial pressure). Furthermore, additional neuroimaging is carried out to identify complications and predisposing conditions associated with BM. Initial radiological imaging for BM should be critically evaluated since predisposing conditions existing in the head and neck regions could be incidentally identified in the images acquired for the initial evaluation of BM

The management algorithm of the initial evaluation of a patient suspected of having BM is shown in Fig. 1. A prompt and accurate diagnosis followed by an empirical antimicrobial treatment is important in patients with suspected BM [9]. Cerebrospinal fluid (CSF) analysis by lumbar puncture allows for the confirmation of BM, and a radiological examination generally plays an auxiliary role in the initial evaluation [1, 9, 10]. Computed tomography (CT) and magnetic resonance (MR) images of the head are used to confirm existing conditions that could be contraindications for a lumbar puncture (i.e., a central nervous system mass, Chiari malformation type I, or other causes of increased intracranial pressure) [9]. Additional neuroimaging can be carried out in order to identify and monitor complications associated with meningitis, such as hydrocephalus, empyema, abscess, cerebritis, venous occlusion, and ischemia. Further, radiological investigations are indicated in patients who show evidence of predisposing conditions that may trigger BM. Predisposing conditions existing in the head and neck regions may be incidentally identified on neuroimaging acquired during the initial evaluation for BM. Therefore, initial radiological findings of patients with suspected BM should be well scrutinized.

## Classification and diagnostic approach for predisposing conditions associated with recurrent BM

The classification and diagnostic approach for each predisposing condition for recurrent BM are shown in Table 1. Predisposing conditions for recurrent BM can be broadly classified into two categories: (1) infection spread from the adjacent tissue to the CSF space and (2) immunodeficiency [7]. The disease entities in the former category are further divided according to whether there is a structural defect between the CSF space and the adjacent tissue. A predisposing condition with a structural defect (59%) could either be congenital (i.e., spinal dermal sinus tract (DST), inner ear malformation, and skull base cephalocele) or acquired (i.e., skull base trauma). Parameningeal infections without a structural defect (5%) primarily include sinusitis, otitis media, and mastoiditis, in which infections can spread continuously through the bony structure of the skull or via the hematogenous route. Immunodeficiency (36%) predisposing to recurrent BM ranges from congenital to acquired diseases [2, 3, 7].

**Table 1:**
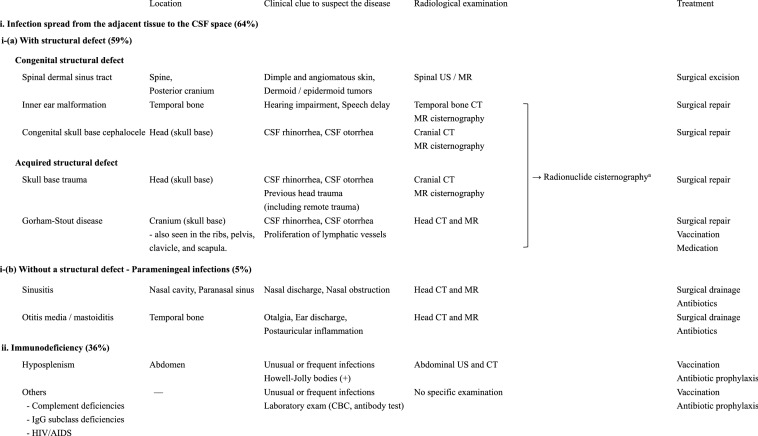
The classification and diagnostic approaches for each condition predisposing patients to recurrent bacterial meningitis

*CBC* complete blood count, *CSF* cerebrospinal fluid, *CT* computed tomography, *HIV/AIDS* human immunodeficiency virus/acquired immunodeficiency syndrome, *IgG* immunoglobulin G, *MR* magnetic resonance, *US* ultrasound

^a^Radionuclide cisternography is used to confirm the cerebrospinal fluid (CSF) leak if the CT and MR imaging results are unable to identify the CSF leak site

Non-radiological examinations, including a patient’s medical history, physical examinations, and laboratory examinations, provide important clinical clues informing the identification of predisposing conditions for recurrent BM [7].

Radiological examinations should be implemented for further assessment once a predisposing condition is suspected after non-radiological examinations. In patients with a suspected DST, spinal ultrasound (US) and MR imaging are useful to visualize associated intraspinal abnormalities (i.e., tethered spinal cord and dermoid/epidermoid tumor) [11, 12]. In patients with suspected sinusitis and otitis media/mastoiditis, CT or MR images may also identify any associated intracranial diseases (subdural/intracranial abscess, venous thrombus) [13, 14]. When a structural defect is suspected in a patient with a CSF leak, CT including CT cisternography or MR imaging are first-line imaging examinations. CT images provide the necessary spatial resolution to reveal even tiny skull base and inner ear malformations or defects. MR cisternography using a steady-state free precession technique allows for multiplanar reformats and facilitates the localization of the actual site of the CSF leak and detection of encephalocele or meningoencephalocele. Herniation of brain parenchyma or meninges through the bone defect could be easily visualized on MR images [15–17]. Invasive radiological examinations, including radionuclide cisternography, should be implemented as a second-line step if the noninvasive images are unable to identify the CSF leak site. Radionuclide cisternography using technetium-99 m-labeled diethylenetriaminepentaacetic acid can diagnose the CSF leak. Radionuclide cisternography involves the intrathecal administration of the radiotracer, followed by acquisition of the images [16]. The accumulation of the radiotracer in the nasal cavity or nasopharynx points to the presence of a CSF fistula [15, 16]. Underlying immunodeficiency is mostly diagnosed clinically, and radiological examination plays a lesser role in this entity compared with the former categories. However, recurrent BM is sometimes accompanied by asplenia that can be diagnosed by abdominal US or CT images.

A treatment plan should be considered for predisposing conditions, along with antibiotic therapy for the BM itself. Surgical intervention should be carried out for diseases with structural defects. Sinusitis or otitis media/mastoiditis accompanying a parameningeal infection should be treated with surgical drainage. Patients with immunodeficiency should receive appropriate vaccinations and prophylactic antibiotics [7].

The following sections describe the detailed clinical features and imaging approach for each disease based on the classifications in Table 1.

## Infection spread from the adjacent tissue to the CSF space with a structural defect: congenital structural defect

### Spinal dermal sinus tract (DST)

#### Clinical features

DSTs are epithelium-lined tracts extending from the skin surface to the intradural space that occur in approximately one in every 2500 live births [18]. DSTs result from the incomplete separation of the cutaneous ectoderm from the underlying neuroectoderm between the third and eighth week of gestation. The tracts may terminate in the dura, spinal cord, conus medullaris, or filum terminale. The tracts are predominantly located in the lumbosacral region and, less commonly, in the occipital region, typically along the midline of the neuroaxis [11, 12, 18].

Recurrent BM may occur due to the spread of the pathogen via the DST from the skin [7, 19]. *E. coli* is the most common causative pathogen for recurrent BM in patients with a DST, most likely due to secondary to fecal contamination into the lumbosacral tract [6]. Inspection of the dermis is useful to assess the presence of a DST. Typical dermal findings in patients with a DST include a large and asymmetric dimple at the ostium that is remote from the anus. The overlying skin may show signs of an angiomatous lesion, abnormal pigmentation, skin tag, and hypertrichosis. Subcutaneous lipomas may be found around the ostium of a DST. If the local infection is associated with a DST, erythema and induration may be observed [12, 18, 19]. Approximately half of all DSTs are associated with dermoid or epidermoid tumors. It has also been noted that aseptic recurrent meningitis can occur due to the rupture of the dermoid or epidermoid into the CSF space [18–20].

#### Management

A DST associated with recurrent meningitis should be managed with appropriate antibiotics therapy followed by surgical excision of the tract [20]. Complete removal of the tract is necessary to prevent the repeated entry of the pathogen into the CSF space. Detethering of the tethered spinal cord and removal of the dermoid or epidermoid are also necessary if they are associated with the DST.

#### Radiological assessment

**Fig. 2:**
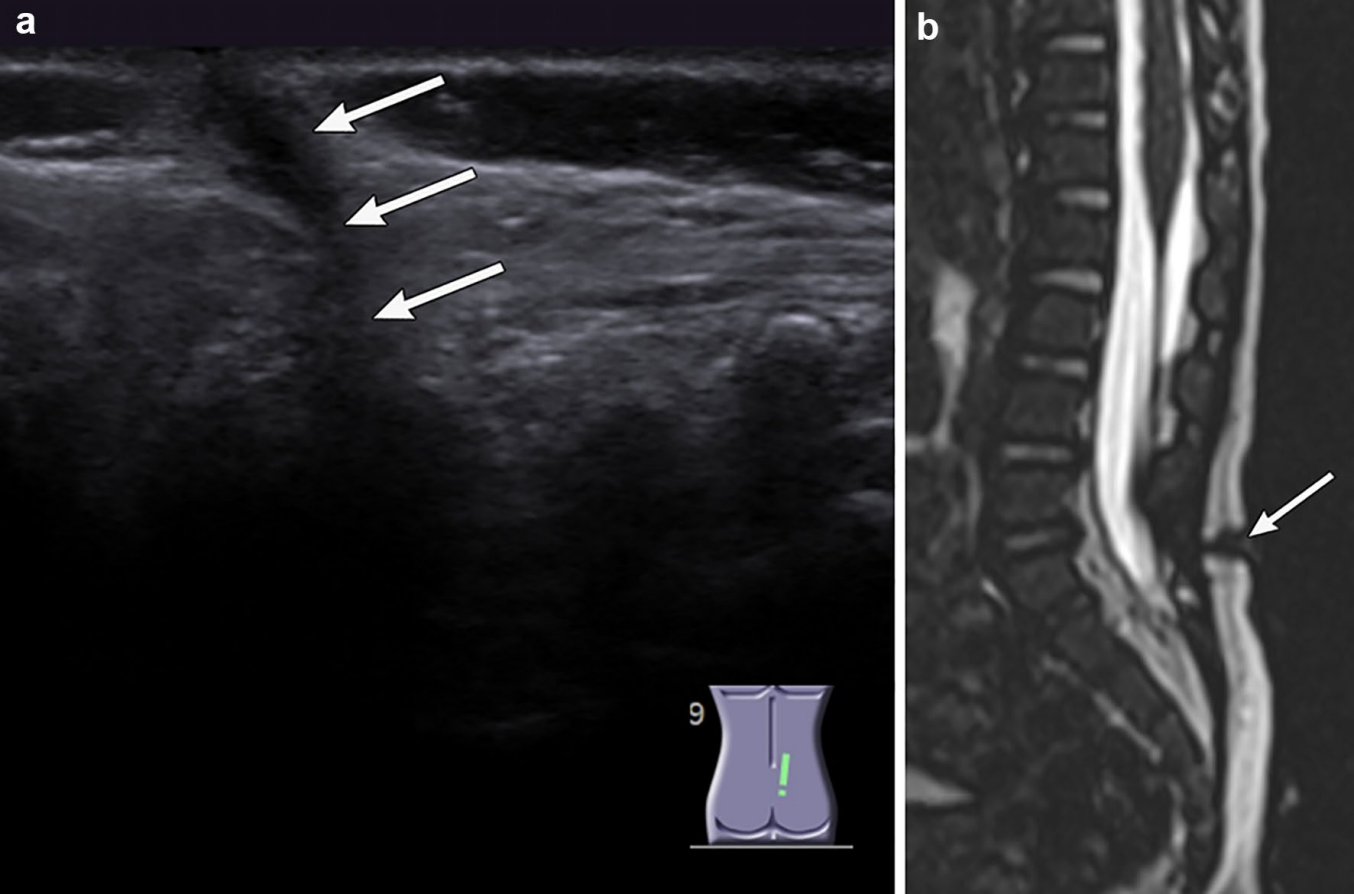
A spinal dermal sinus tract (DST) in a 1-year-old boy with bacterial meningitis (BM). A sacral dimple with cutaneous stigmata was found at 1 month. Initial spinal ultrasound (US) performed for screening purposes at 1 month did not detect any obvious abnormalities. The patient was admitted due to BM, and spinal US was performed again when the patient was 1 year old. **a** US at age 1 year shows a continuous tract to the spinal canal from the skin (white arrows). **b** Sagittal T2-weighted magnetic resonance image clearly shows a DST connected to a skin dimple from the dural sac (white arrow)

A spinal US can be performed as a first-line examination for the evaluation of the spinal cord in newborns or infants under the age of 6 months with a DST to visualize the tract and intraspinal lesion [12, 19, 21, 22]. Spinal MR imaging is superior in detectability for not only the DST itself but also its associated anomalies compared to the spinal US. Therefore, spinal MR imaging should be performed when a physical examination indicates the potential presence of a DST [11, 21] (Fig. 2).

A low conus medullaris is observed in 79% of patients with a DST. Intraspinal tumors, including spinal lipoma, dermoid and epidermoid, are associated with a DST. Dermoid and epidermoid tumors are commonly located at the intraspinal end of tracts, although they can be found at any location along with the DST [18].

**Fig. 3:**
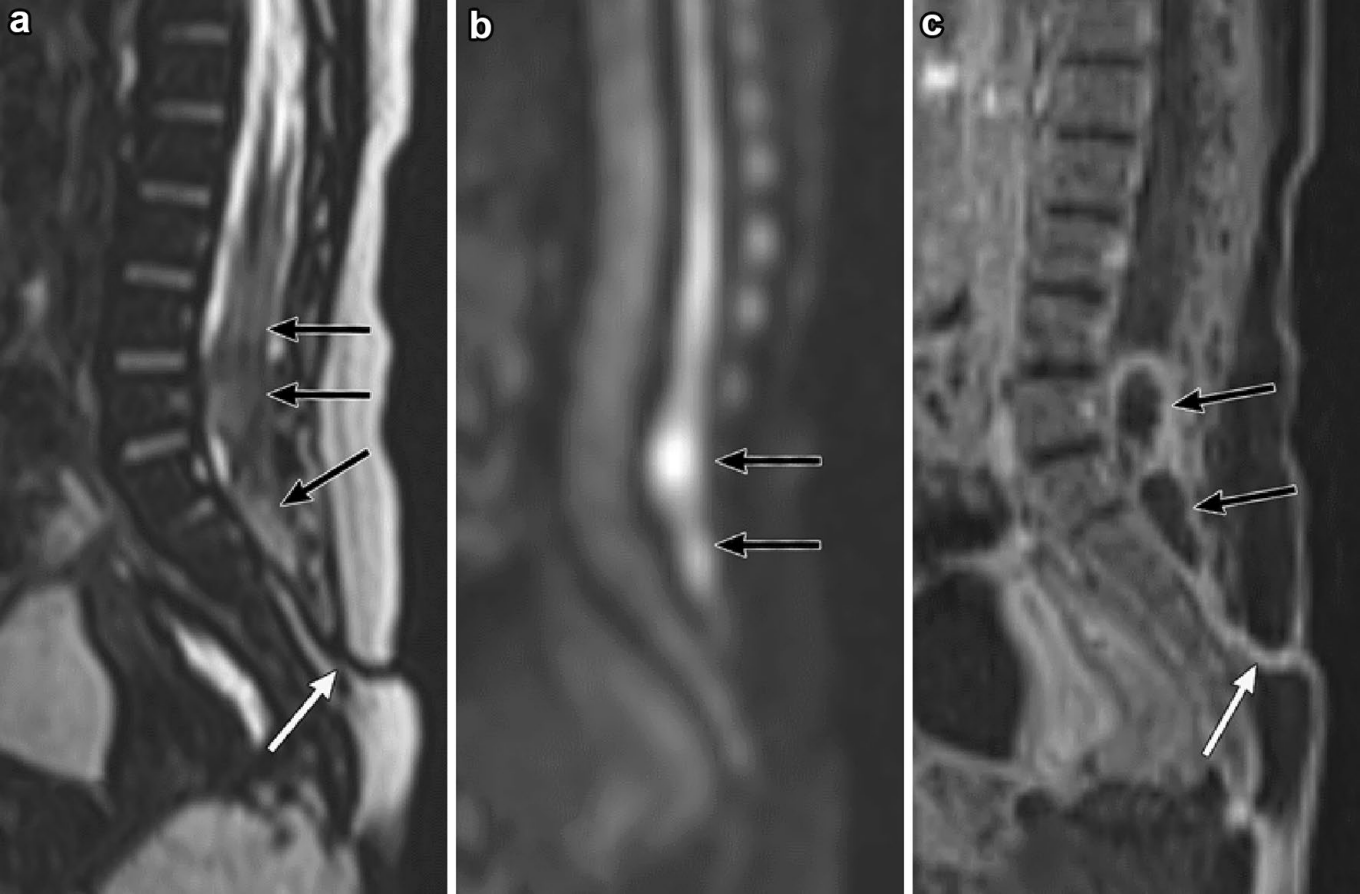
A spinal dermal sinus tract (DST) in a 3-month-old girl with bacterial meningitis (BM). A sacral dimple was first noted during her hospitalization for BM. **a** Sagittal T2-weighted magnetic resonance (MR) image shows a low conus medullaris. The caudal end of the spinal cord is tethered by a DST (white arrow). The spinal cord is thickened, and hyperintense tumor-like structures are observed in the dural sac (black arrows). **b** Diffusion-weighted image shows the tumor-like structures with diffusion restriction in the dural sac (black arrows). **c** Gadolinium-enhanced fat-suppressed T1-weighted MR image shows that the tract (white arrow) and rim of the tumor-like structures (black arrows) are enhanced. The differential diagnoses at this point include an infected dermoid or epidermoid and intraspinal abscess in the dural sac. Surgical resection of the DST and untethering of the spinal cord were performed. An intraspinal abscess and a dermoid tumor in the dural sac were also resected during surgery

Gadolinium-enhanced spinal MR imaging is useful to detect inflammatory changes associated with a DST [23, 24]. The peripheral enhancement of the tract and spinal cord can be observed in meningitis associated with the DST (Fig. 3). An intramedullary and extramedullary abscess may sometimes accompany BM. An intraspinal abscess can be seen as a peripheral enhancing mass lesion and can mimic infected dermoid or epidermoid tumors (Fig. 3). In addition, both entities show strong diffusion restriction. Therefore, radiologists should be aware that an underlying intraspinal tumor may be missed on MR imaging in patients with an intraspinal infection. In such cases, it is recommended to take a follow-up MR image to confirm the presence of the tumor after the meningitis is resolved.

### Inner ear malformation

#### Clinical features

Congenital inner ear malformations result from a genetic disorder or a maternal infection during fetal development [25, 26]. Inner ear malformations are classified based on the time of developmental arrest of the inner ear, and the severity of the disease is correlated with the time of arrest. Various inner ear malformations may result from developmental arrest between the third and seventh gestational week [27]. A type I incomplete partition resulting from the cochlear development arrest in the fifth week is less well differentiated than a type II incomplete partition (Mondini deformity) that results from developmental arrest in the seventh week [27]. A type II incomplete partition is the most common type of cochlear malformation, accounting for 50% of all cochlear deformities [27, 28]. A type II incomplete partition is characterized by a cochlea with a normal basal turn and cystic apex and an enlarged vestibule. A type I incomplete partition shows a separated cystic cochlea and vestibule [27].

Congenital inner ear malformations account for 15% of all patients with recurrent BM [7]. The most common pathogen responsible for BM is *S. pneumoniae* in patients with inner ear malformations. Initial episodes of BM due to inner ear malformations often occur in the first decade of life [29]. Recent widespread use of newborn hearing screening has improved the rate of early diagnosis of inner ear malformations that can cause sensorineural hearing loss. In some cases, however, hearing loss remains unrecognized and recurrent BM can lead to a diagnosis of inner ear malformation [7, 30]. BM itself can cause a temporary hearing impairment that could be a potential differential diagnosis for underlying inner ear malformations [7, 31].

**Fig. 4:**
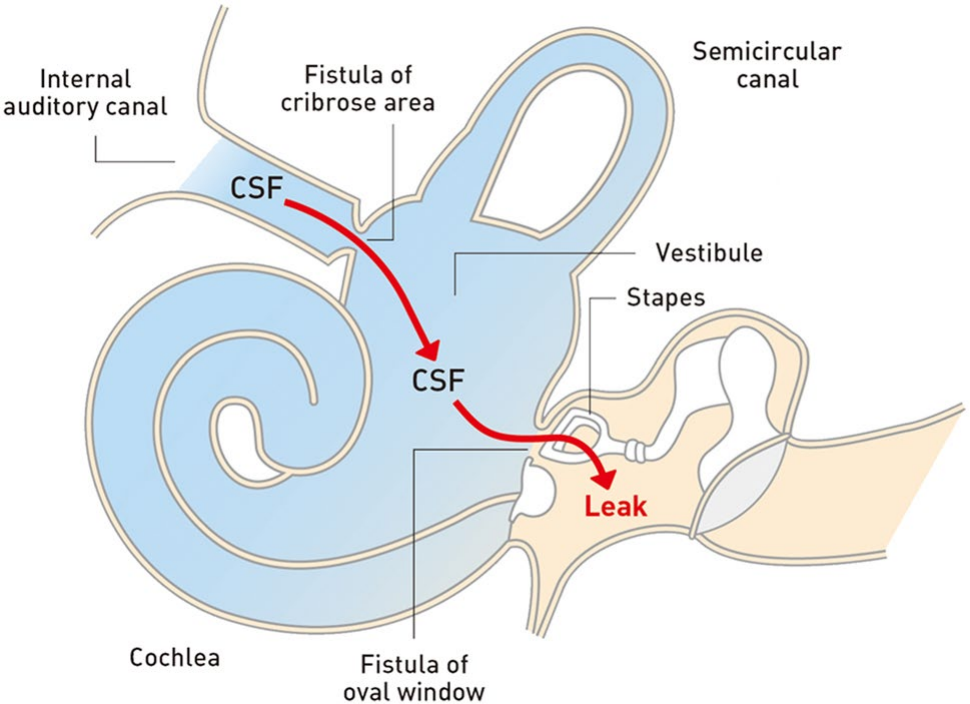
Illustration of pathways of the internal auditory canal to external auditory canal fistulas. Bacterial meningitis results from the two fistulous connections: (1) between the internal auditory canal and the inner ear and (2) between the inner ear and middle ear. It is likely that a fistula of the cribriform area is a site of cerebrospinal fluid (CSF) leak, in the medial side of the inner ear in patients with an inner ear malformation. CSF leak to the middle ear from the inner ear can occur at the oval window due to the fistula in the region of the stapes footplate

BM results from two fistulous connections: (1) between the internal auditory canal and the inner ear and (2) between the inner ear and the middle ear (Fig. 4). It is likely that a fistula of the cribriform area between the cochlea and internal auditory canal is a site of CSF leak in the medial side of the inner ear in patients with inner ear malformations. CSF leaks into the middle ear from the inner ear, can occur at the oval window due to the fistula in the region of the stapes footplate [27, 32, 33]. Patients with a cochlear implant are known to have an increased risk of BM due to the iatrogenic connection between the inner and middle ears. A CSF leak is a more common surgical complication in cases of type I incomplete partition (64%) than in cases of type II incomplete partition (37%) [26, 34].

#### Management

Surgical closure of the fistula from the subarachnoid space to the middle ear is the definitive treatment for the prevention of recurrent BM [29]. Pneumococcal vaccinations are also useful in preventing recurrent meningitis, although surgical treatment is indicated [31].

#### Radiological assessment

**Fig. 5:**
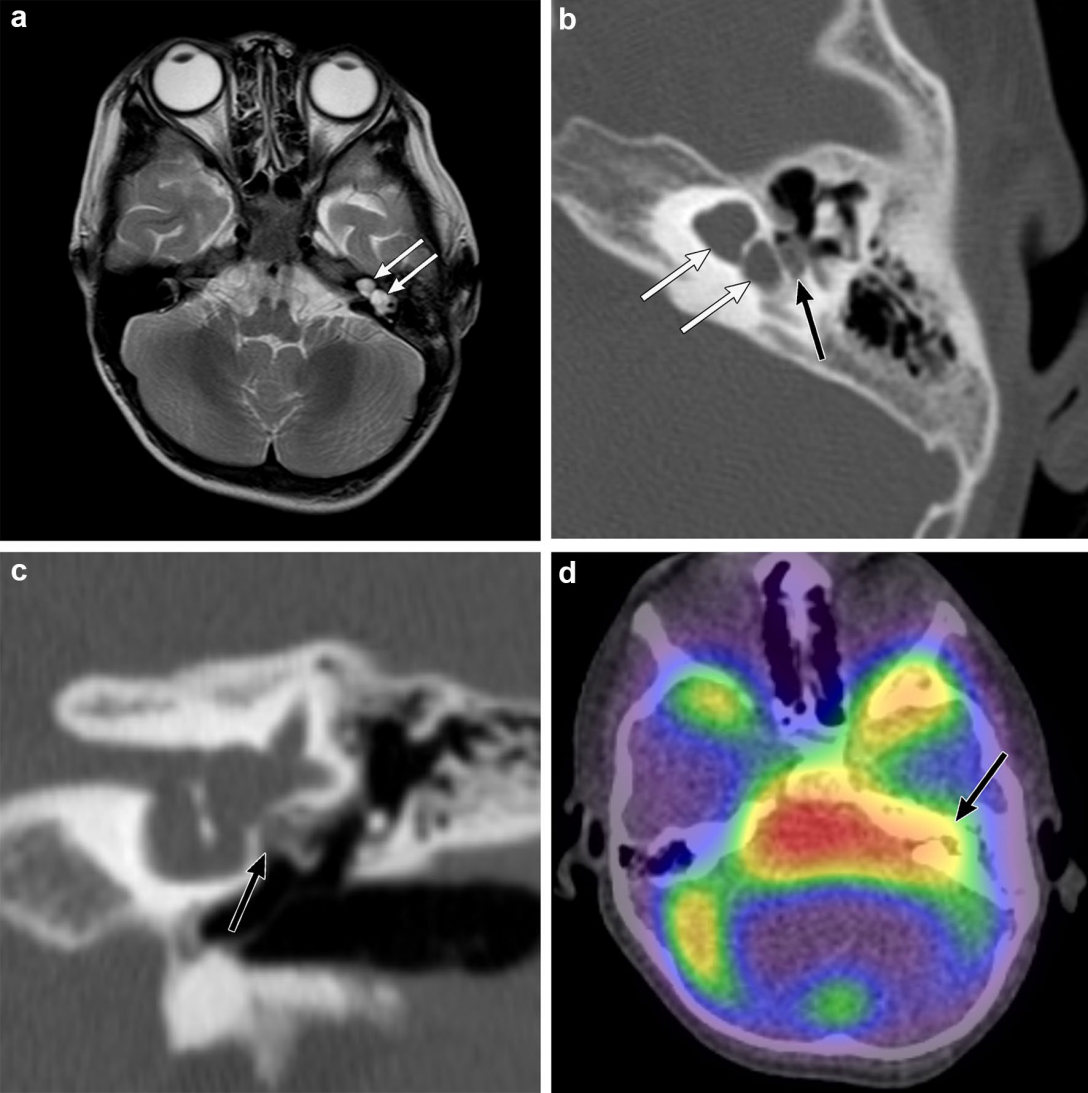
Left inner ear malformation (incomplete partition type I) in a 5-year-old boy with recurrent bacterial meningitis. The patient has left sensorineural hearing loss. **a** T2-weighted magnetic resonance image shows that the enlarged cochlea and vestibule in the left temporal bone suggest an inner ear malformation (white arrows). **b** Axial computed tomography (CT) of the temporal bone shows that the left cochlea has a cystic appearance and the vestibule is enlarged and cystic (white arrows). The cochlea and vestibule are separated, suggestive of incomplete partition type I. Fluid accumulation is observed in the lateral side of the left oval window, highly suggestive of a cerebrospinal fluid (CSF) leak (black arrow). **c** Coronal CT of the temporal bone shows fluid accumulation protruding through the left oval window (black arrow), suggestive of a CSF leak through a fistula in the left oval window associated with inner ear malformations. **d** Radionuclide cisternography with a Single-photon emission CT/CT fusion image shows increased radiotracer accumulation in the left temporal bone (black arrow), and a CSF leak into the left middle ear is confirmed. Leak of CSF from a partial defect of the stapes footplate is confirmed during the surgical repair

CT and MR imaging of the temporal bones are used to reveal and assess inner ear malformations [27, 28, 34]. CT with thin slice, multi-planar reconstruction of the temporal bone is required to evaluate inner ear malformations. CT and MR imaging can help distinguish subtypes and the severity of inner ear malformations. A CSF leak through the oval window can be observed in the region of the stapes footplate. Fluid protruding through the oval window on CT and MR imaging strongly suggests the presence of a fistula through the oval window (Fig. 5) [32]. Fluid accumulation in the middle ear and mastoid cells is also useful as an indirect finding linked to a CSF leak. Radionuclide cisternography is able to reveal the CSF leak as reflected in the accumulation of the radiotracer in the inner and middle ears. Single-photon emission computed tomography (SPECT)/CT fusion imaging in radionuclide cisternography is a useful technique for more accurate localization of CSF leak sites than planar images (Fig. 5) [35].

### Congenital skull base cephalocele

#### Clinical features

Congenital skull base cephalocele is a herniation of intracranial contents through a skull base defect with an incidence of about 1:40,000 [36]. Congenital skull base cephaloceles might remain unrecognized at birth since the herniated content exists within the nasal or paranasal cavity and cannot be observed on physical examination. Skull base cephalocele can cause airway obstruction in the nose, nasopharynx, or oropharynx [36–38].

**Fig. 6:**
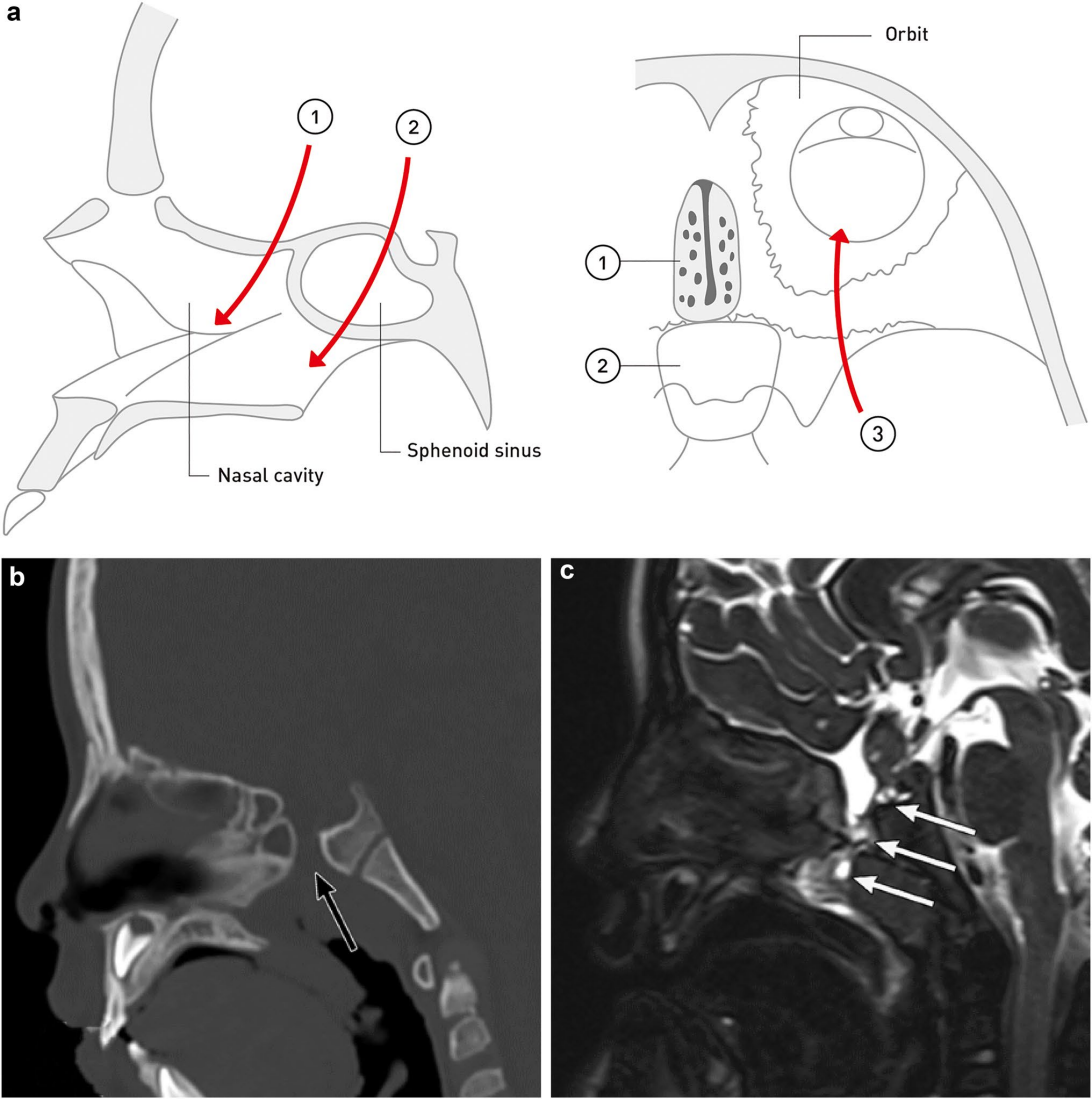
Congenital skull base cephalocele. **a** Classification of congenital skull base cephalocele. Skull base cephaloceles are classified into (i) transethmoidal, (ii) transsphenoidal, and (iii) spheno-orbital. The arrows indicate the pathway by which intracranial contents protrude into the extracranial space through a skull base defect. **b** Congenital skull base cephalocele (transsphenoidal cephalocele) in a 5-year-old boy with bacterial meningitis and growth hormone deficiency. Sagittal head computed tomography shows a partial defect of the sphenoid bone (black arrow). **c** Sagittal T2-weighted magnetic resonance image shows herniated contents extended into the nasopharynx through the defect of the sphenoid bone (transsphenoidal cephalocele). Herniated content shows heterogenous intensity, with fluid components showing high intensity, and herniated brain parenchyma and dysplastic gliotic tissue showing iso intensity (white arrows). The pituitary gland has a deformity and is herniating into the sphenoid bone defect

Congenital skull base cephaloceles can be classified into three different types according to the location: (1) transethmoidal cephalocele, (2) transsphenoidal cephalocele, and (3) spheno-orbital cephalocele [10, 37] (Fig. 6a). The pituitary gland, optic nerve, and hypothalamus commonly show deformities and herniation through the skull base defect in patients with transsphenoidal cephalocele, and visual disturbance and pituitary hypothalamic dysfunction can also be observed [36–38]. Transsphenoidal cephalocele is also associated with other congenital malformations, including holoprosencephaly, corpus callosum agenesis, and midfacial anomalies such as a cleft lip [37, 38].

Recurrent BM associated with a CSF leak from a skull base osseodural defect can occur in either type of congenital skull base cephalocele. *S. pneumoniae* is the most common causative organism of BM in patients with a congenital skull base cephalocele. A fiberoptic endoscopy is useful to observe a CSF leak and a herniated cephalocele in the nasal cavity.

#### Management

Surgical repair should be performed to prevent the recurrence of BM. The safety and efficacy of endoscopic endonasal approaches have been reported in recent years [39].

#### Radiological assessment

CT demonstrates the congenital osseous defect, continuous with the herniated contents, entering into the adjacent paranasal sinus or nasal cavity [16, 36]. MR imaging would be useful to discriminate among the components of the herniated sac (i.e., CSF, brain tissue, or dysplastic gliotic tissue) (Fig. 6c). Scrutinizing CSF continuity between the herniated contents and subarachnoid space may be helpful in distinguishing skull base cephaloceles from intra-nasal tumors. MR imaging is also useful to visualize morphological abnormalities in adjacent structures, including the optic nerve, pituitary gland, and vascular structures [16, 36].

## Infection spread from the adjacent tissue to the CSF space with a structural defect: acquired structural defect

### Skull base trauma

#### Clinical features

CSF leaks occur in approximately 2% of head injuries and in about 10–30% of skull base fractures [40, 41]. A CSF leak from a skull base trauma most commonly involves the anterior cranial fossa, resulting from fractures of the frontal sinus or cribriform plates of the ethmoid bones [15, 40]. In 80% of post-trauma patients, a CSF leak spontaneously resolves within 48 h after the occurrence of the skull base fracture. In patients with a persistent CSF leak after head trauma, the leak site usually becomes apparent within the first month, but some may remain unrecognized until several years after the trauma [7, 40–42]. BM occurs in 7–30% of patients with a persistent CSF leak after a head trauma [40]. The causative organisms of BM in patients with a skull base fracture are *S. pneumoniae* and *H. influenzae*. Skull base trauma is the most common cause of recurrent BM associated with anatomical problems [7].

A detailed history of recent and remote traumatic events is essential to form a diagnosis. Rhinorrhea and otorrhea are common clinical symptoms in patients with a CSF leak. It should be noted that CSF rhinorrhea sometimes remains unrecognized [7]. Further investigations, including β2-transferrin testing, fiberoptic endoscopy, and otoscopy, are required if either CSF rhinorrhea or otorrhea is suspected [7].

#### Management

In the acute post-traumatic phase, most CSF leaks resolve with conservative treatments such as bed rest and head elevation [16]. Patients with persistent CSF leaks due to skull base fractures require surgical repair since these patients are at a higher risk of recurrent BM [16, 40, 41]. Other effective treatments include CSF tap and lumbar drain placement, which could reduce intracranial pressure and facilitate healing. Appropriate vaccinations are required especially in patients who are not indicated to receive surgical repair [7, 43].

#### Radiological assessment

**Fig. 7:**
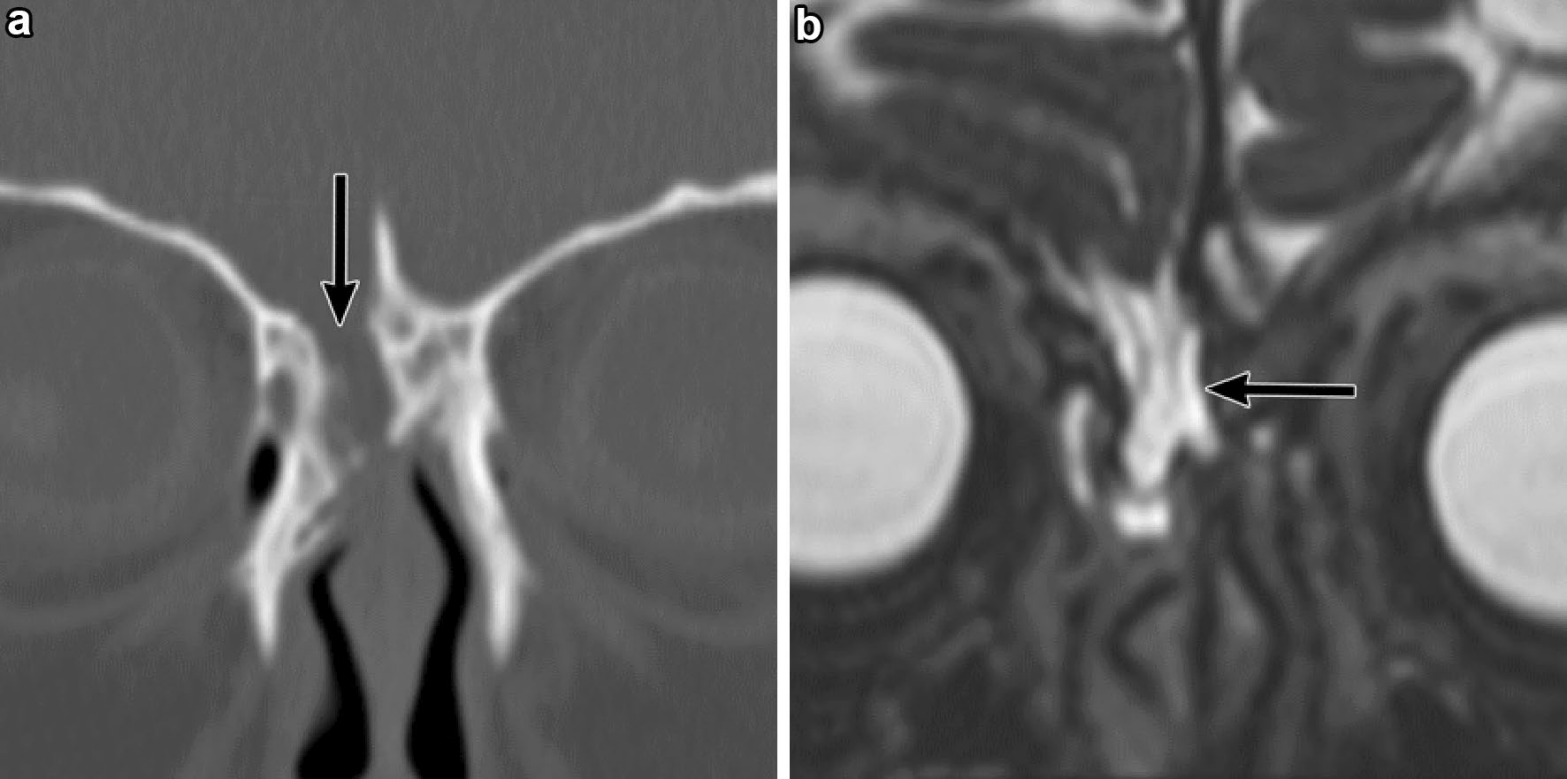
Multiple skull base fractures in a 3-year-old boy with recurrent bacterial meningitis. The patient had a head injury in a traffic accident 2 years prior to presentation. **a** Coronal computed tomography shows that the cerebrospinal fluid space communicates with the ethmoid antrum through a partial defect of the cribriform plate (black arrow). **b** Magnetic resonance (MR) cisternography (coronal T2-weighted MR image) shows the meningocele (black arrow) protruding through a defect of the cribriform plate. Although not shown here, there were also fractures in the left temporal bone

CT imaging can reveal skull base fractures causing CSF leaks. Identifying the anatomic location of the CSF leak is essential for surgical repair. Skull base fractures responsible for the CSF leak are sometimes very small and should be carefully evaluated with thin slice CT with multiplanar reconstruction [15–17]. Coronal CT images are required to assess fractures in the ethmoid roof and planum sphenoidale that are almost never apparent on axial CT images (Fig. 7).

**Fig. 8:**
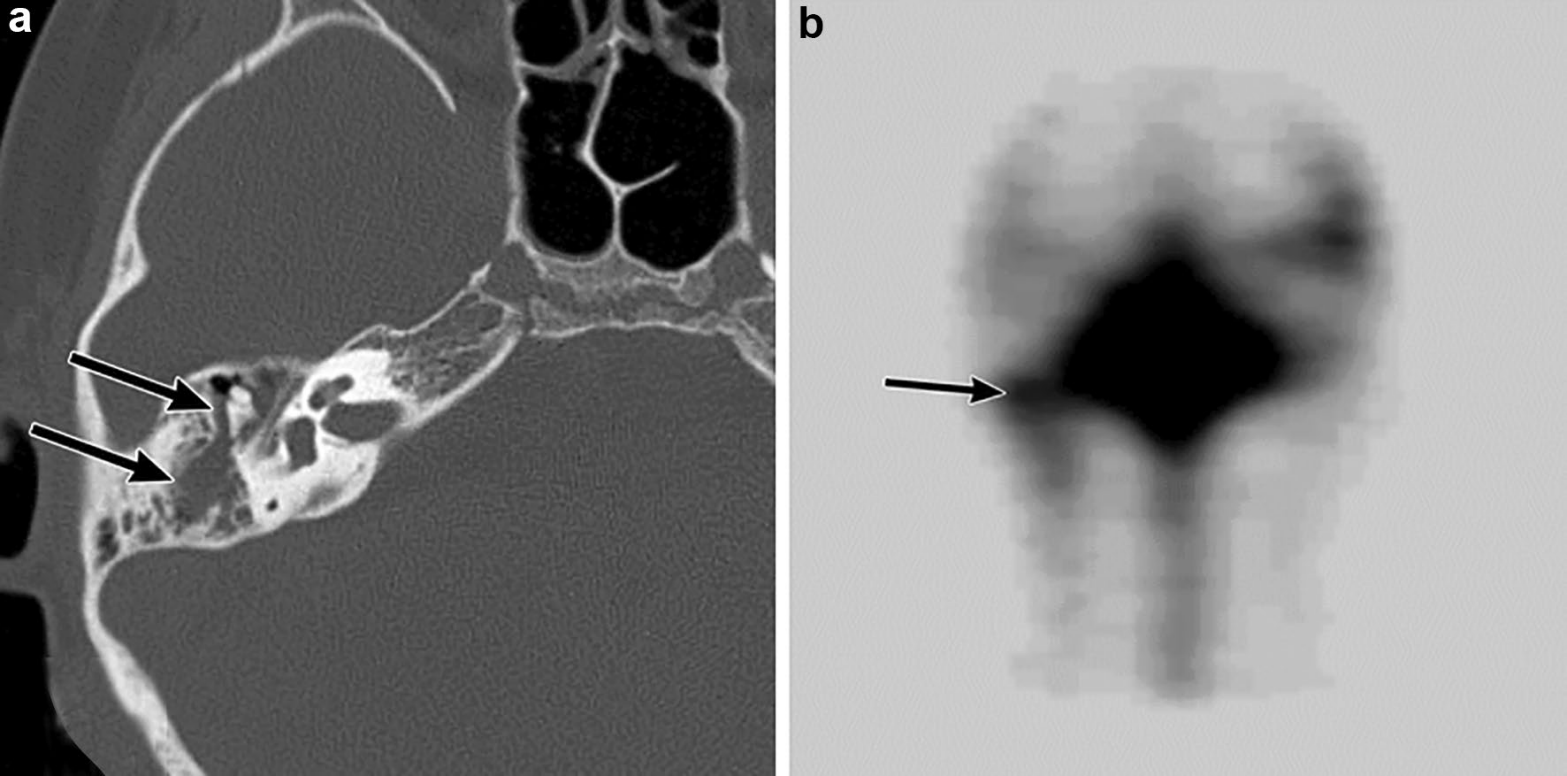
Cerebrospinal fluid (CSF) leak in the right middle ear in a 10-year-old boy who was diagnosed with bacterial meningitis five times. The patient had a head injury 8 years prior to presentation. **a** Axial temporal bone computed tomography shows fluid collection in the middle ear and mastoid air cells, suggestive of a CSF leak (black arrows). It is probably caused by remote head trauma, but fractures cannot be identified. **b** Radionuclide cisternography shows radionuclide accumulation in the right mastoid cells and the right middle ear, and a CSF leak was confirmed (black arrow). A lumbar-peritoneal shunt was performed to prevent the CSF leak. Surgical repair was not performed since the exact leak site remained unclear

MR imaging is useful for detecting CSF leaks and is indicated to discriminate meningoencephalocele, which contains brain parenchyma and meninges in the herniated content, from meningocele, which only contains meninges in the herniated content [15, 16, 44–46]. MR cisternography is a good indication for the localization of the CSF leak site in patients with multiple bone defects identified on CT scans [15]. Fluid accumulation in the middle ear, mastoid air cells, and paranasal sinuses can be an indirect sign of a CSF leak (Fig. 8).

Radionuclide cisternography is used to confirm a CSF leak if the CT and MR images cannot identify the CSF leak site. Radionuclide cisternography is limited to detecting CSF leaks that are active at the time of the study. Delayed image acquisition, up to 24–72 h after the administration of the radiotracer, could be beneficial for detecting intermittent CSF leaks [15, 16].

### Gorham–Stout disease

#### Clinical features

Gorham–Stout disease, also known as massive osteolysis or vanishing bone disease, is a rare disorder characterized by progressive osteolysis. Pathologically, the disease is characterized by the proliferation and dilation of lymphatic vessels [47, 48]. Gorham–Stout disease can affect any part of the skeleton, but is more common in the ribs, cranium, pelvis, clavicle, and scapula. Pathologic fractures and skeletal pain are common symptoms [48]. Patients with craniofacial involvement of Gorham–Stout disease are at risk of CSF leaks, and some patients with temporal bone involvement suffer from BM due to CSF leaks [49–52]. BM may sometimes lead to a diagnosis of Gorham–Stout disease [51, 52].

#### Management

Several medications, including bisphosphonates, bevacizumab, and interferon-alpha 2b, have been used to treat patients with Gorham–Stout disease. A recent clinical trial is testing the efficacy of sirolimus [53]. Bone loss with functional impairment requires surgical resection and reconstruction [54]. Surgical repair of the skull base has been reported to be effective in terminating CSF leaks [50].

#### Radiological assessment

**Fig. 9:**
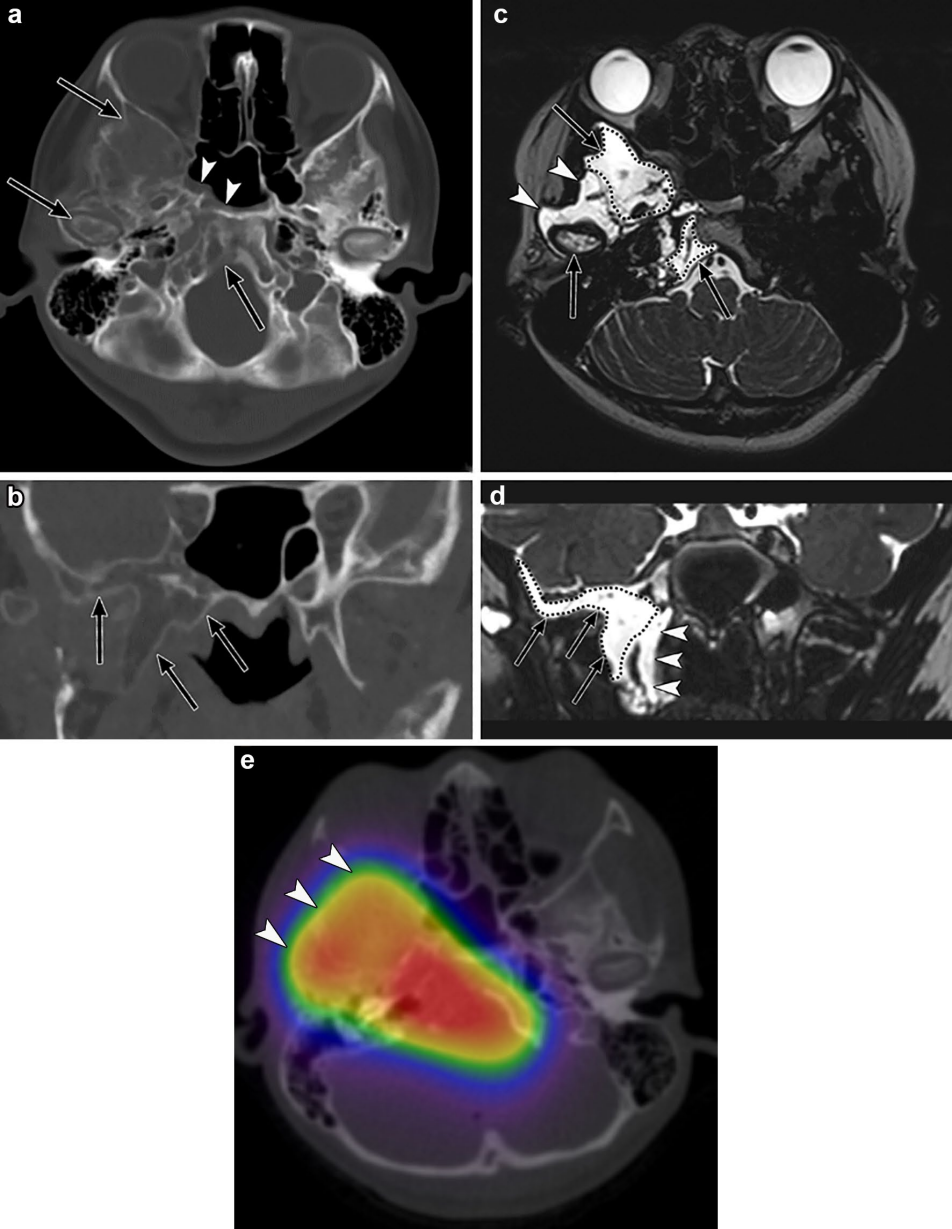
Gorham–Stout disease in a 12-year-old girl with recurrent bacterial meningitis. The chief complaint was right neck pain and trismus. **a**, **b** Axial and coronal head computed tomography (CT) (bone window) show infiltrative osteolytic changes and expansion of the bone in the right skull base bone. The sphenoid bone and the right mandibular condyle are involved (black arrows). Fluid accumulation is observed in the sphenoid sinus (arrowheads). **c**, **d** Axial and coronal head magnetic resonance images (T2-weighted images) show that the right skull base is extensively involved and appears hyperintense (black arrows). Fluid accumulation is observed in the parapharyngeal space, suggestive of a cerebrospinal fluid (CSF) leak (arrowheads). Note that it is difficult to distinguish involved bones from a CSF leak because both show similar high intensities on T2-weighted images. **e** Single-photon emission CT/CT fusion imaging in radionuclide cisternography shows radionuclide accumulation in the right cervical fascial space, and a CSF leak is confirmed (arrowheads)

Gorham–Stout disease is characterized by cortical resorption and progressive, often extensive, osteolysis on radiographs and CT scans (Fig. 9). Gorham–Stout disease begins with lucencies in the intramedullary or subcortical regions, sparing the cortex [48]. Therefore, early bony lesions in Gorham–Stout disease are difficult to identify. CSF leaks should be suspected in Gorham–Stout disease with osteolysis of the skull base, especially the temporal bone, and fluid accumulation in the middle ear, the mastoid air cells, or fascial spaces.

MR imaging is useful to assess osteolytic lesions as well as CSF leaks. Bony involvements in Gorham–Stout disease show up as hyperintensities on T2-weighted images, similar to CSF, reflecting the lymphatic nature of this disorder (Fig. 9) [55]. Therefore, it may be difficult to identify CSF leaks in patients with Gorham–Stout disease. Radionuclide cisternography is useful to confirm CSF leaks in Gorham–Stout disease, as it is able to distinguish CSF leaks from a lymphatic anomaly (Fig. 9) [50].

## Infection spread from the adjacent tissue to the CSF space without a structural defect: parameningeal infections

### Sinusitis

#### Clinical features

Sinusitis is defined as an inflammation involving the paranasal sinuses mucosa. Common pathogens involved in bacterial sinusitis are *Streptococcus pneumoniae*, *Haemophilus influenzae*, and *Moraxella catarrhalis* [56]. Bacterial sinusitis is associated with not only BM but also other intracranial complications, including brain abscesses, epidural empyema, and dural sinus thrombosis. Intracranial infections associated with sinusitis have been reported in 3% of hospitalized pediatric patients [57]. The frontal sinuses are most commonly associated with sinogenic intracranial infections [58]. The infection usually occurs via the progression of the septic thrombi through the cranial valveless diploic veins that penetrate the dura or, less commonly, through the direct intracranial extension of osteomyelitis [58–60].

Patients with sinusitis usually present with a purulent nasal discharge, cough, fever, headache, and nasal congestion. These symptoms sometimes remain unrecognized in children until their sinusitis worsens [61]. Therefore, initial radiological imaging for BM should be evaluated carefully to look for the presence of incidental sinusitis.

#### Management

Antimicrobial therapy is of primary importance in the management of intracranial complications. Surgical drainage of the paranasal cavity is sometimes required [59, 62]. Neurosurgical intervention to drain the intracranial abscess is required if it is associated with sinusitis.

#### Radiological assessment

**Fig. 10:**
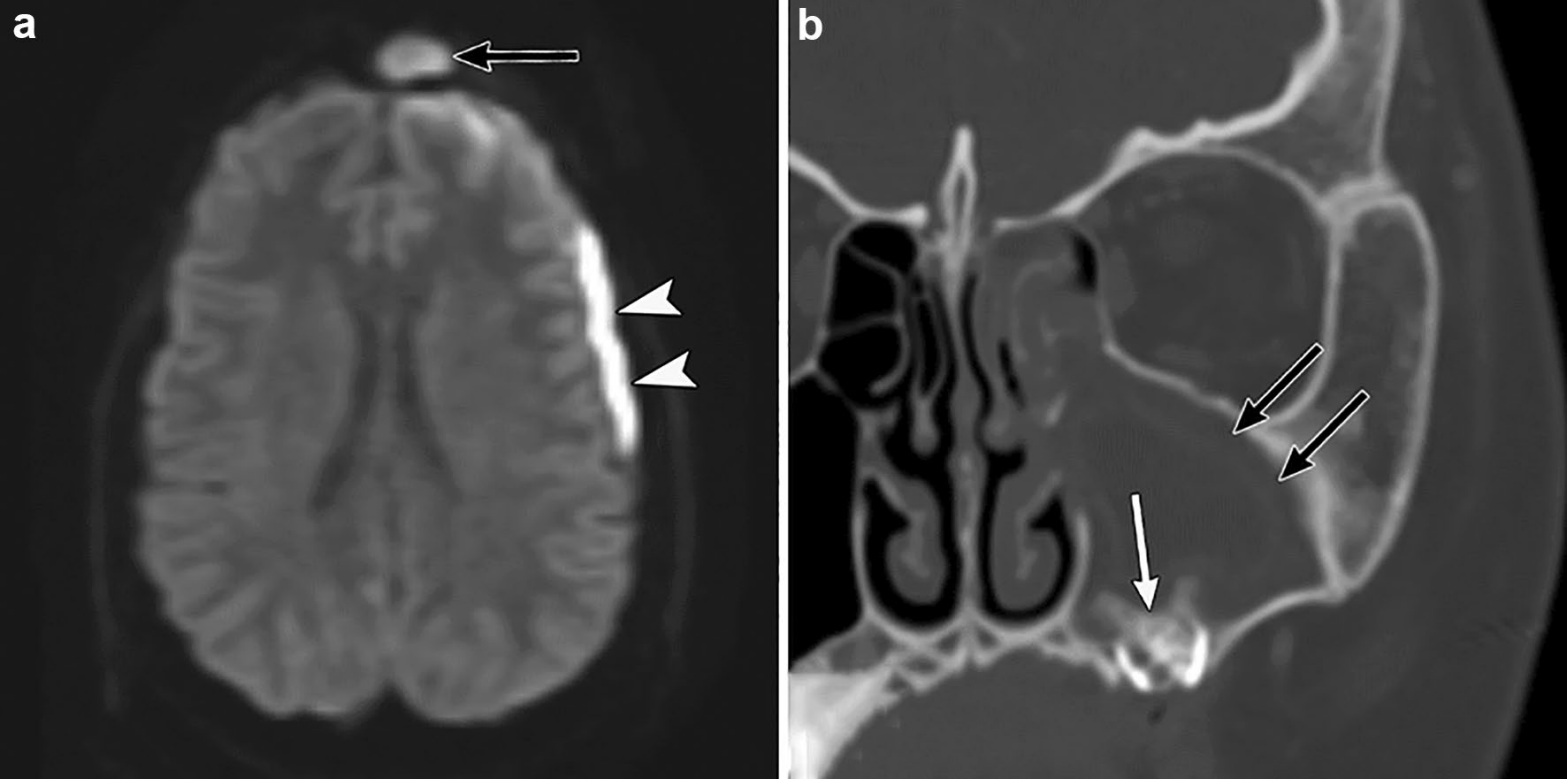
Odontogenic sinusitis in a 12-year-old girl with bacterial meningitis. **a** Diffusion-weighted image shows subdural fluid accumulation with diffusion restriction, suggestive of subdural empyema (arrowheads). Restricted diffusion is also observed in the frontal sinus (black arrow). **b** Coronal computed tomography (bone window) shows the left maxillary sinus with massive fluid accumulation and mucosal thickening (black arrows). Impacted decaying teeth are observed in the left maxillary sinus (white arrow), confirming the diagnosis of odontogenic sinusitis. Surgical drainage for subdural empyema and endoscopic sinus surgery were performed. The impacted teeth were extracted

CT and MR imaging findings of sinusitis include mucosal thickening, fluid accumulation, and mucosal enhancement. These findings are non-specific and do not necessarily reflect the severity of the symptoms. Therefore, imaging for acute, uncomplicated sinusitis is not usually recommended [14, 58, 63]. Where intracranial complications associated with sinusitis are suspected, CT with contrast enhancement and/or MRI with or without contrast enhancement, including the paranasal sinuses and cranium, is recommended. Contrast-enhanced MR imaging is superior to contrast-enhanced CT imaging in detecting meningitis and other intracranial complications [58]. Diffusion-weighted imaging can reveal suppurative material as it typically shows restricted diffusion (Fig. 10) [58]. In unilateral sinusitis, underlying odontogenic causes or the presence of foreign bodies in the paranasal cavity should also be evaluated (Fig. 10).

### Otitis media and mastoiditis

#### Clinical features

Acute otitis media, an inflammation of the middle ear, is the most common infection during the first 5 years of life [13]. Acute mastoiditis, a serious complication of acute otitis media, also mainly occurs in young children. *S. pneumoniae* and group A beta-hemolytic *streptococcus* are the most common pathogens for BM in patients with acute otitis media. Infections spreading to the bony structure cause osteomyelitis. Infections spreading beyond the temporal bone cause intracranial complications, including BM, intracranial abscesses, and vascular thrombosis. Epidural abscesses and sinus thrombosis are common complications associated with mastoiditis in children [13]. Symptoms of otitis media and mastoiditis include otalgia, ear discharge, and retroauricular inflammation [64]. In coalescent mastoiditis, the demineralization of bone septa and osteonecrosis of the thinner mastoid walls result in the creation of large purulent cavities [13].

#### Management

A mastoidectomy should be performed in patients with intracranial complications [64]. Coalescent mastoiditis usually requires surgical drainage. Therefore, early detection of coalescent mastoiditis by radiological imaging is crucially important [14].

#### Radiological assessment

**Fig. 11:**
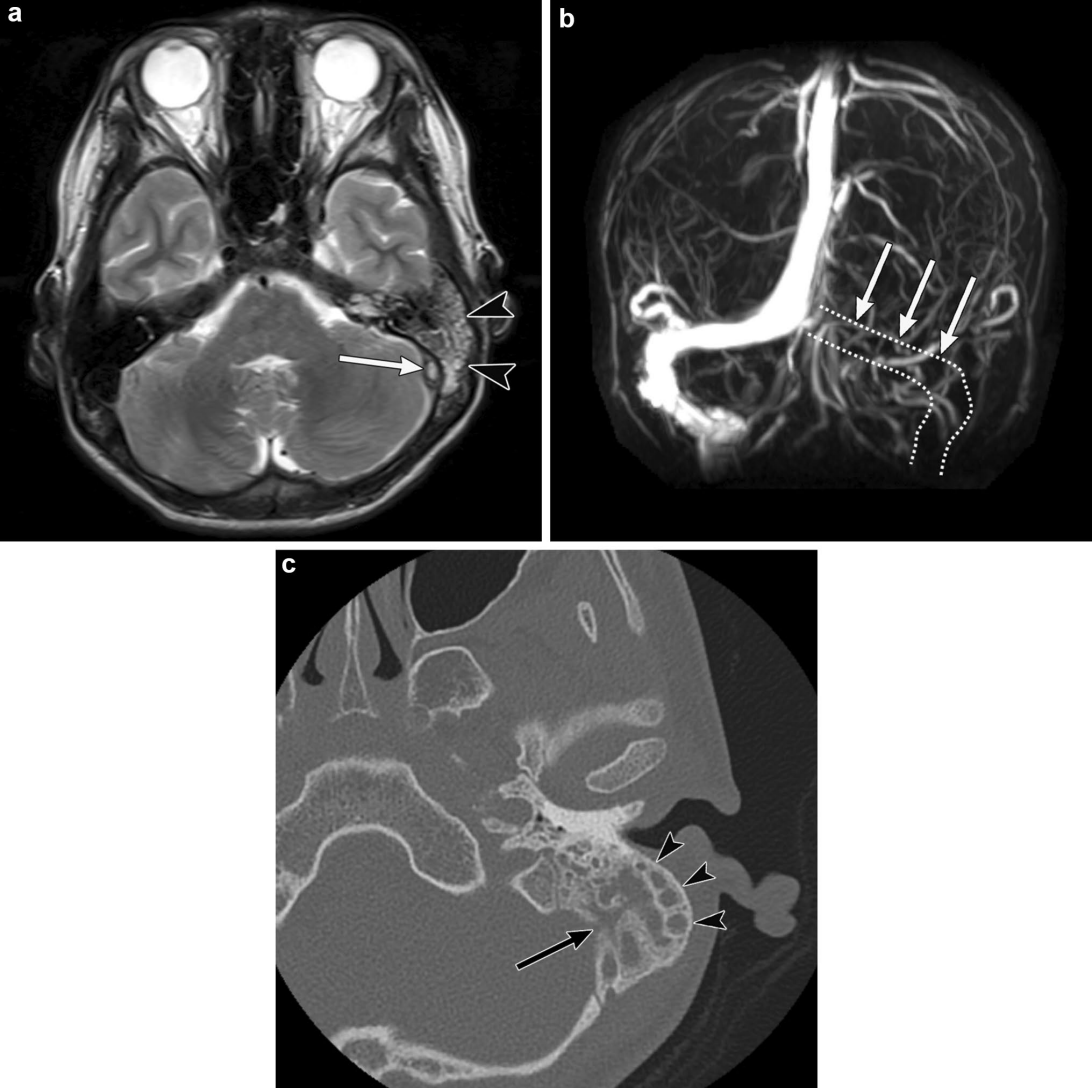
Mastoiditis in a 13-year-old boy with bacterial meningitis. **a** Axial T2-weighted magnetic resonance (MR) image shows fluid collection in the left mastoid bone (arrowheads). The left sigmoid sinus shows the absence of a flow void, suggestive of sinus thrombosis (white arrow). **b** Maximum-intensity projection of a contrast-enhanced MR venogram shows a complete thrombosis of the left sigmoid sinus to the left transverse sinus (white arrows). **c** Axial computed tomography (bone window) shows fluid accumulation in the left mastoid air cells (arrowheads). The medial mastoid wall shows lytic changes (black arrow)

CT and MR images show fluid accumulation in the middle ear and the mastoid air cells. CT imaging shows erosion of the mastoid septa or mastoid wall in patients with coalescent mastoiditis (Fig. 11) [13, 14]. Patients with coalescent mastoiditis are more likely to have intracranial infections and venous thrombosis than those with mastoiditis without bone destruction [14]. The absence of flow void in spin-echo T2-weighted images points to the presence of dural venous thrombosis (Fig. 11). Dural venous thrombus can be observed as a low-intensity intravascular structure on T2-weighted gradient-echo MR images. Contrast-enhanced CT and contrast-enhanced MR are generally required to make a definitive diagnosis of dural venous thrombosis. Contrast-enhanced MR venography depicts dural venous thrombosis better than non-contrast time-of-flight MR venography since the image quality is affected by the turbulent flow in the vessels [13, 65].

## Immunodeficiency

### Hyposplenism

#### Clinical features

Hyposplenism is defined as the impairment of splenic function, and is associated with several disorders such as sickle-cell anemia, bone marrow transplantation, graft-versus-host disease, and splenic morphological abnormalities including polysplenia and asplenia [66]. The spleen plays an important role in immunological functions. Antibody production is important to remove encapsulated bacteria, including *S. pneumoniae*, *H. influenzae*, and *Neisseria meningitides* [66]. Patients with hyposplenism are at an increased risk of severe infections with encapsulated bacteria, including recurrent BM [7, 66–69]. Both asplenia and polysplenia can cause hyposplenism, leading to severe bacterial infections, including meningitis [70, 71]. A history of previous abdominal trauma or surgery should raise the suspicion of hyposplenism [7]. The detection of Howell-Jolly bodies, erythrocytes with nuclear remnants, is a useful technique for screening for hyposplenism [66].

#### Management

Appropriate vaccinations and antibiotic prophylaxis are required for patients with splenic dysfunction to prevent severe infections, including BM [70].

#### Radiological assessment

**Fig. 12:**
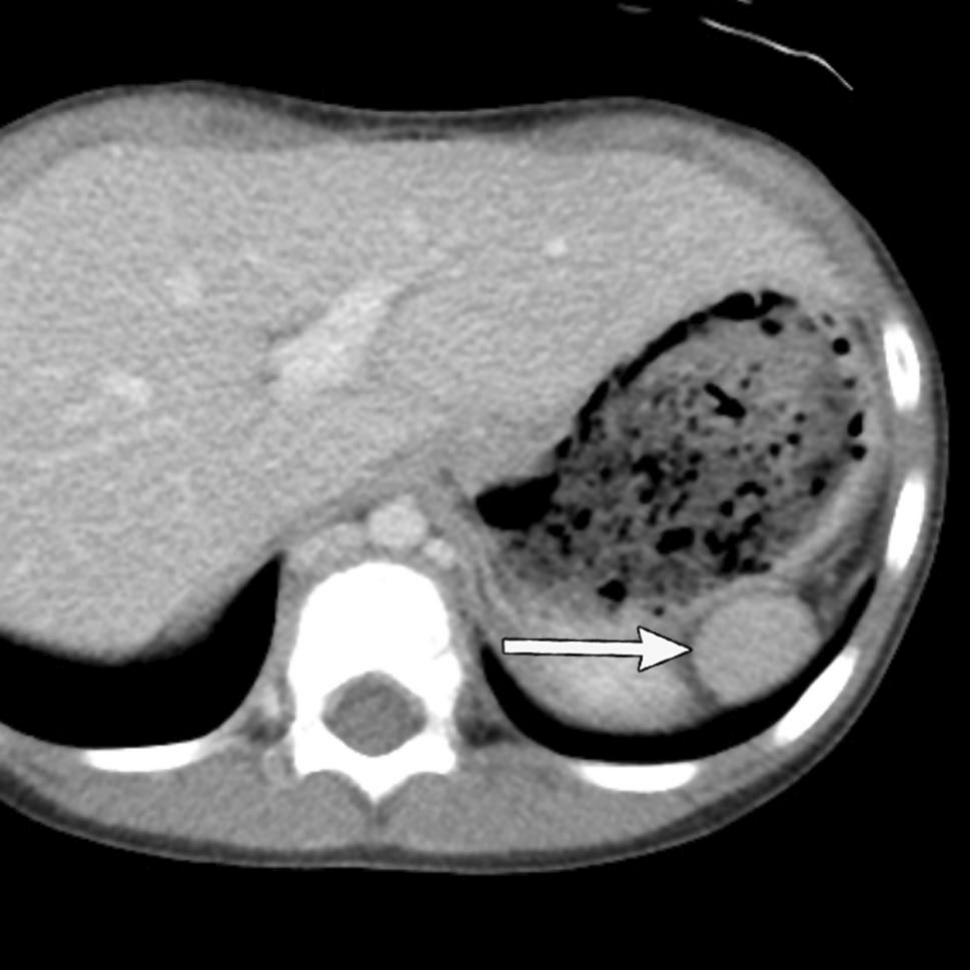
Hyposplenism in a 1-year-old boy with bacterial meningitis. Howell–Jolly bodies and target cells were confirmed. Contrast-enhanced computed tomography imaging confirmed the presence of a small singular spleen without a parent spleen (white arrow)

Abdominal US or CT imaging can allow for the visualization of the morphology and the size of spleen (Fig. 12). In patients with congenital asplenia or polysplenia, an underlying heterotaxy syndrome and associated complex congenital anomalies should also be assessed. The malformations including complex congenital heart disease and midgut malrotation that can influence prognosis must be carefully evaluated on imaging [71, 72].

## Conclusion

One of the roles of the radiologist in managing BM is to diagnose predisposing conditions. Predisposing conditions for recurrent BM can be broadly classified into two categories: infection spread from the adjacent tissue to the CSF space and immunodeficiency. Diseases in the former category are further divided according to whether there is a structural defect between the CSF space and the adjacent tissue. Knowledge of the clinical features and imaging findings linked to these predisposing conditions and an appropriate radiological approach may enable early diagnosis, treatment, and prevention of recurrent BM.

## Data Availability

Not applicable.
